# Comparison and optimization of DNA Isolation protocols for high throughput genomic studies of *Acacia pachyceras* Schwartz

**DOI:** 10.1016/j.mex.2022.101799

**Published:** 2022-07-28

**Authors:** Nazima Habibi, Fadila Al Salameen, Muhammed Rahman, Anisha Shajan, Farhana Zakir, Nasreem Abdulrazzack

**Affiliations:** Environment and Life Science Research Centre, Kuwait Institute for Scientific Research, Shuwaikh Campus, Kuwait

**Keywords:** DNA, Next-generation sequencing, Tree species

## Abstract

We describe the optimization and validation of six DNA isolation protocols from fresh leaves of the rare tree *Acacia pachyceras*. The first four protocols employed three commercial kits (Sigma, Nucleospin1, Nucleospin 2, Promega) whereas the remaining two were based on the traditional sodium dodecyl sulfate (SDS) and cetyltrimethylammonium bromide CTAB methods. Each protocol provided significantly different results concerning DNA concentration (*p* < 0.032), yield (*p* < 0.000), contaminant carry over, protocol duration, cost per sample, and comprehensive cost. We demonstrated the applicability of all the tested protocols in DNA barcoding. The protocol yielded maximum amounts (92.85 µg) of DNA in a rapid turnaround time (8 h). The quantity and purity surpassed all the other tested methods. DNA extracted by the CTAB method was the best for NGS (Phred score >Q30). These protocols will be useful tools for molecular research of *Acacia pachyceras* and other closely related tree species.

## Graphical abstract

**Figure:**
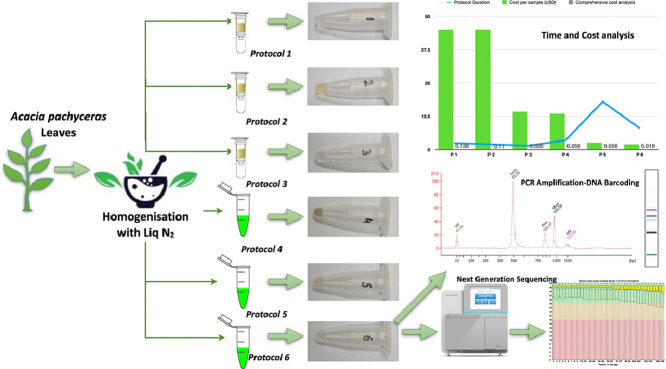

Specifications Table

**Table:** 

**Subject Area:**	Biochemistry, Genetics and Molecular Biology
**More specific subject area:**	Plant Molecular Biology
**Protocol name:**	DNA Extraction protocol
**Reagents/tools:**	Nucleospin, Macherey Nagel, Cedex, FranceGen-Elute Plant Genomic DNA Miniprep Kit, Sigma Aldrich, Darmstadt, GermanyWizard genomic DNA purification kit, Promega, Madison, WI, USATris, Gibco BRL, ultra-pure, Life Technologies, CA, USEDTA, Sigma Aldrich, NY, USAProteinase K, (10 mg/ml), Qiagen, MD, USASDS, Gibco BRL, Life Technologies, CA, USPotassium acetate, Sure Chem products, EnglandIsopropanol, Carlo Erba Reagents, FranceEthanol, Merck, Honeywell Raedel-de-Haen FrancePVP, Sigma Aldrich, GermanyHydrochloric Acid, Merck, Darmstadt, GermanyPotassium metabisulfite, BDH Laboratories, EnglandDTT, Sigma Aldrich, GermanyNaCl, BDH Laboratories, EnglandCTAB Bioworld Fine Research Chemicals, OH, USANaCl, BDH Laboratories, England2-mercaptoethanol, Merck, Darmstadt, GermanyChloroform, Fisher Scientific, UKIsoamyl alcohol, Merck, Darmstadt, GermanyRNase A, Qiagen, USAHOT FIREPol^Ⓡ^ Blend Master mix, Solis BioDyne, EstoniaPrimers Thermo Fisher Scientific (Waltham, MA, USA)matK X F- 5′ TAATTTACGATCAATTCATTC’ matK 5r R- 5′ GTTCTAGCACAAGAAAGTCG 3′trnH- F- 5′CGCGCATGGTGGATTCACAATCC 3′ psbA-R- 5′ GTTATGCATGAACGTAATGCTC 3′ rbcL-a F- 5′ ATGTCACCACAAACAGAGACTAAAGC 3′ ajf634 R- 5′ GAAACGGTCTCTCCAACGCAT-3′InstrumentsVeriti, Thermocyclers, Applied Biosystems, Grand Island, NYQubit, Fluorormeter, Invitrogen, WAAgarose Gel Electrophpresis System, BioRadChemidoc MP, BioRad, USAUV–vis Spectrophotometer, Nanodrop, ThermoFischerBioanalyzer 2100, Agilent, Darmstadt, GermanyHiSeq 2500, Illumina, San Diego, US
**Experimental design:**	Fresh leaf tissue was crushed to a fine powder in liquid nitrogen. Three commercial kits namely the GenElute Plant Genomic DNA Miniprep kit (Sigma Aldrich, Darmstadt, Germany), NucleoSpin Plant II, Mini kit (Macherey-Nagel, Cedex, France) and Wizard Genomic DNA purification kit (Promega, Madison, WI, USA) and two conventional protocols popularly known as SDS (sodium dodecyl sulfate) and a CTAB (cetyl trimethyl ammonium bromide) method were used for DNA extraction. All the isolations were performed in duplicate under strictly aseptic conditions. A comprehensive time and cost per sample were estimated for each method. DNA extracted by the above methods was quantified on a Nanodrop UV/Vis spectrophotometer (ThermoFisher). The ratios at A260/280 nm and A230/260 nm were recorded. The DNA concentrations were also estimated on a fluorometer (Qubit, Invitrogen, WA) employing the BR dsDNA assay kit. The universal matK, rbcL, and trnF were used to check the applicability of the extracted DNA in PCR. High-quality DNA obtained by the CTAB method was used for next-generation sequencing
**Trial registration:**	NA
**Ethics:**	NA
**Value of the Protocol:**	• The protocol provides a baseline for an issue of primary importance in molecular research of polyphenol and polysaccharide-rich plant tissue. • This method is usable in low-technology laboratories for sample preparation and is suitable for various molecular analytical techniques and amplification of plant barcode genes (matK, rbcL, TrnF). • The resulting optimized CTAB protocol enables the isolation of high-quality genomic DNA amenable to next-generation sequencing in the genus Acacia and related species. • The protocol will be of value to plant biologists working with hardy tree species in the area of population genetics, conservation biology and biodiversity management.

## Description of protocol

The loss of biodiversity in arid lands due to the harsh climatic conditions is an issue of global concern [1]. The first critical steps in protecting and managing threatened species are to investigate their ecological and evolutionary characteristics through genetic studies [2], [3]. Various molecular methods ranging from the traditional restriction enzyme-based or polymerase chain reaction (PCR) based molecular markers [4], [5], [6] to the most advanced next-generation sequencing (NGS) have become valuable tools in all aspects of conservation genetics [7], [8], [9]. Where traditional genome analysis relies on relatively small amounts of deoxyribonucleic acid (DNA) of moderate purity, on the other hand, NGS technologies require the input of several micrograms of high molecular weight DNA [10]. Therefore, the extraction of DNA in good qualities and quantities is of utmost importance in developing these studies [11], [12], [13], [14]. *Acacia pachyceras* Schwartz. an endemic tree species in Kuwait is on the verge of extinction [15], [16]. High throughput genomic research is largely lacking in the economically important genus, Acacia. The species is extremely rich in polysaccharide and polyphenol content, which hampers good quality DNA isolation. The CTAB protocol in this study was used for NGS based genome survey analysis of *A. pachyceras* [10]. The data generated was mined for thousands of microsatellite motifs that will eventually be used for genetic diversity studies of this endangered species.

## DNA extraction

Young leaf samples were snipped off the branches and immediately placed in polythene bags, appropriately labelled and transported on ice to the Kuwait Institute for Scientific Research (KISR) laboratories. The leaves were aliquoted in small sample size and stored at −20 °C until further use. The frozen leaves were weighed and crushed to a fine powder in liquid nitrogen in an autoclaved mortar and pestle. All the isolations were performed in triplicates.

## Protocol I-GenElute plant genomic DNA Miniprep Kit (Sigma Aldrich)

A total of 100 mg, of ground plant tissue, was added to 350 µL of Lysis Solution [Part A] and 50 µL of Lysis Solution [Part B] were added to the tube and mixed thoroughly by vortexing and inverting, followed by the addition of 50 units of RNase A. The mixture was incubated at 65 °C for 10 min with occasional inversion to dissolve the precipitate. Post incubation, 130 µL of Precipitation Solution was added to the mixture and placed on ice for 5 min. The lysate was centrifuged at 12,000 g for 5 min to pellet the cellular debris, proteins, and polysaccharides. The supernatant from this step was carefully pipetted to a filtration column and centrifuged for 1 min. Thereafter, 700 µL of Binding Solution was added to the flow-through and mixed thoroughly. In the meantime, the binding column was prepared by adding 500 µL of the Column Preparation Solution and centrifugation at 12,000 g for 1 min. The lysate from the previous step was loaded onto the binding column and centrifuged at 12,000 g for 1 min. For washing the bound DNA, 500 µL of the wash solution was added to the spin column and centrifuged at maximum speed for 1 min. This step was repeated twice and followed by dry centrifugation to get rid of any remaining flow through. The DNA was finally eluted in 50 µL of pre-warmed (65 °C) Elution Solution. The elution buffer was added to the center of the column and centrifuged at maximum speed for 1 min.  The elution was repeated twice to ensure complete dissolution of DNA.

## Protocol II-NucleoSpin plant II, Mini kit (Macherey-Nagel)

Two extraction buffers PL1 (CTAB based) and PL2 (SDS based) are available in this kit and both methods were employed for DNA extraction in the present investigation. The amount of plant material taken was double the recommended amounts and therefore the lysis solution and buffers were increased proportionately.

A total of 200 mg of plant powder was transferred to a clean tube. As per the instructions provided in the instruction manual, 800 µl of lysis buffer PL1 and 20 µL of RNase A (50 U) were added to the first set of samples and incubated at 65 °C for 10 min. The lysate was transferred to a spin column for filtration by centrifugation at 11,000 x g for 2 min. Approximately 900 µL of PC was added to the flow-through, and the contents were transferred to the DNA binding column, where it was centrifuged for 1 min at 11,000 x g. Subsequently, the bound DNA was washed once with 800 µL of PW1 and twice with 1400 and 400 µL of PW2, respectively. The spin columns were centrifuged in between each washing step at 11,000 g for 1 min. The final elution of DNA was done in 50 µL of pre-warmed (65 °C) PE (5 mM Tris–HCl, pH 8.5). A double elution was done with the same column.

## Protocol III- NucleoSpin Plant II, Mini kit (Macherey-Nagel)

Similar steps to protocol II were repeated with the second set of samples by replacing the buffer PL1 with 600 µL of PL2 followed by the addition of 150 µL buffer PL3 (Potassium acetate) and incubation on ice for 5 min. All the remaining buffers/solutions were increased proportionately as recommended in the kit. Elution was done twice to maximize yield.

## Protocol IV-Wizard Genomic DNA purification kit (Promega)

In this protocol, 1 ml of Nuclei lysis solution was added to 500 mg of ground tissue and incubated at 65 °C for 15 min. The lysate was subjected to RNase treatment by adding 3 µL of RNase solution and incubating at 37 °C for 15 min. The mixture was cooled at room temperature and 200 µL of protein precipitation solution was added. After incubating for 5 min on ice the solution was centrifuged at 13,000 g for 3 min. The upper aqueous layer was carefully transferred to a fresh tube containing 1 vol of isopropanol. The sample was gently mixed by inversion and DNA was allowed to precipitate by centrifugation at 13,000 g for 1 min. The supernatant was discarded and washed with 70% ethanol (1 vol). After washing the purified DNA was vacuum dried for 15–20 min and dissolved in 100 µl of TE buffer [17]. Prior to dissolution the TE buffer was pre-warmed at 65 °C. The DNA was left in TE buffer overnight at 4 °C to ensure complete dissolution.

## Protocol V-SDS method

Approximately 1.5 g  of plant tissue was added to 30 ml of extraction buffer (100 mM Tris, 50 mM EDTA, 500 mM NaCl, 50 mM DTT, 20 mM Potassium metabisulfite, pH:8.0) [18], [19]. The lysate was vortexed thoroughly before adding 2 g of PVP (Polyvinylpyrrolidone, 10,000). The entire solution was revortexed and allowed to reach room temperature. Thereafter, 5 µl of RNase (10 mg/ml) was added and incubated in a water bath for 60 min at 37 °C. Following the RNAse treatment, proteinase K (final concentration of 100 µg/ ml) was added and left overnight at 37 °C. The next day, 3 ml sodium dodecyl sulfate (SDS; 20% stock) was added, mixed well and incubated at 65 °C for 15 min followed by the addition of 12 ml of 5 M potassium acetate. Mixed and incubated at 0 °C (ice bucket) for 20–30 min. (final conc. of 5 M potassium acetate is 2%). Centrifugation was done at 15,000 rpm for 20 min. at 4 °C and the supernatant was transferred to a clean tube. Treatment with SDS and Potassium acetate was repeated thrice and added to 0.6 vol of isopropanol to precipitate the DNA. After mixing gently the solution was incubated at −20 °C for 30 min and then centrifuged at 17,500 rpm for 30 min. to pellet the DNA. The DNA pellet was washed with 70% ethanol twice by centrifugation at 10,000 rpm for 10 min at RT. The pellet was air-dried overnight and dissolved in TE buffer. The list of individual reagents used in the protocol is provided in Table S1.

## Protocol VI–CTAB method

1 g of leaf tissue was ground in liquid nitrogen to a fine powder and transferred to clean oak ridge tubes [20]. 15 ml of prewarmed CTAB buffer (100 mM Tris-Cl, pH 8.0; 1.4 M NaCl; 25 mM EDTA, pH 8.0; 2% CTAB; ß-mercaptoethanol and water to make up the volume) was added to the powdered leaves and incubated at 60 °C for 1 h with intermittent mixing by gentle inversion. Thereafter added 12 ml of chloroform-isoamyl alcohol (24:1v/v) and mixed thoroughly. The lysate was centrifuged at 10,000 g for 10 min at room temperature. The upper aqueous layer was transferred into a new tube with a wide bore pipette. This was followed by the addition of 0.6 vol of isopropanol. The tubes were mixed by gentle inversion to precipitate the DNA. The clear visible white DNA was hooked out with a bent fused Pasteur pipette and transferred to a clean tube. The pellet was washed twice with 70% ethanol leaving at room temperature for 10–15 min between each wash and centrifuging at 10,000 g for 10 min. The final wash was discarded and the pellet was left for air-drying overnight. The dried DNA pellet was dissolved in 15 ml of TE buffer and processed for RNase treatment. To the above 100 ul of 4 mg/ul RNase was added and incubated at 37 °C for 1 h. The extracted DNA was stored at −20 °C until further processing. The list of individual reagents used in the protocol is provided in Table S2.

Modifications to the original CTAB protocol by Doyle and Doyle 1987- Polyvinylpyrrolidone was replaced by ß-mercaptoethanol and phenol: chloroform: isoamyl alcohol by chloroform: isoamyl alcohol.

## DNA purity and quantity estimation

DNA extracted by the above methods was quantified on a Nanodrop UV/Vis spectrophotometer (ThermoFisher). The ratios at A260/280 nm and A230/260 nm were recorded. The DNA concentrations were also estimated on a fluorometer (Qubit, Invitrogen, WA) employing the BR dsDNA assay kit. These concentrations were used to estimate the total yield of DNA. The intactness of DNA was checked on a 0.8% agarose gel in 1X TAE buffer ran at 100 V for 45 min and visualized on a gel doc system (BioRad, CA). One-way analysis of variance and Tukey's post hoc tests were performed in Microsoft Excel for DNA concentration and yield, and means were compared at confidence intervals of 95% and 99%, respectively. A comprehensive time and cost per sample were estimated for each method as described by Wang et al. [21]. (estimated cost per extraction of any one method/maximum estimated cost among eight methods) × (estimated time per extraction of any one method/maximum estimated time among eight methods). The cost of individual reagents used in the CTAB and SDS methods are given in Tables S1 and S2.

**Fig. 1:**
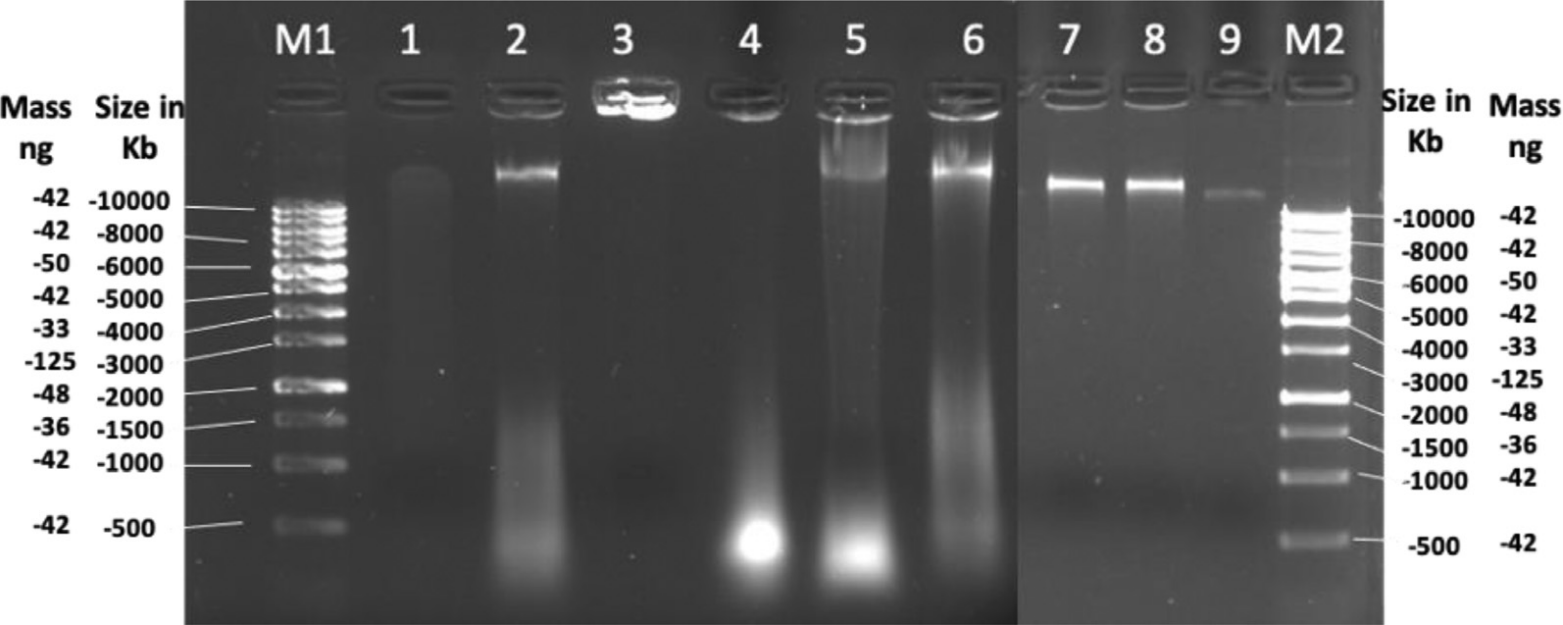
DNA isolated by five different protocols and visualized on 0.8% Agarose gel ran at 10 V/cm for 1 h. M1 and M2–1Kb Marker; DNA isolated by Lane1-Sigma kit; Lane 2-Nucleospin kit protocol 1; Lane 3-Nucleospin kit protocol 2; Lane 4-Promega kit; Lane 5 & 6-SDS method; Lane 7–9-CTAB method.

Each protocol provided significantly different results concerning DNA concentration (*p* < 0.032), yield (*p* < 0.000), contaminant carry over, protocol duration, cost per sample, comprehensive cost and its utility in the high throughput molecular research (Table 1). In terms of yield, the conventional methods outperformed the commercial kits. A significantly higher (Tuckey's *p* < 0.01) yield of ∼92.85 ± 24.8 µg was obtained by the CTAB method followed by SDS (15.28 ± 2.99 µg) > Promega (0.241 ± 0.02 µg) > Sigma (0.216 ± 0.05 µg) > Nucleospin 2 (0.181 ± 0.11 µg) > Nucleospin 1 (0.124 ± 0.15 µg). The desired UV/Vis spectrophotometer absorbance peaks (A260/280 nm = 1.8) were only obtained through the CTAB method. Sigma protocol provided comparable levels of purity (A260/280=1.7), whereas, lower A260/280 ratios of 1.19 (Nucleospin 1), 1.45 (Nucleospin 2), 1.38 (Promega) and 1.33 (SDS), were obtained thru other protocols. The A230/260 nm was very low (0.06–1.26) with all the protocols except CTAB (1.51). The time duration for the six methods ranged from 1.5 to 18 h. The SDS method (18 h) was the most time-consuming followed by the CTAB method (8 h), Promega (3 h), Nucleospin 1 (2.5 h), and Nucelospin 2 (2.0 h). The Sigma kit based (1.5 h) method was the shortest among all the tested protocols. Concerning the cost, the conventional methods (SDS and CTAB) were the cheapest costing approx. 2.0–2.5 USD per sample as compared to the commercial kits costing between 3.6 −20.0 USD for one extraction. When time and cost were considered together CTAB was the most economical followed by the Sigma protocol. The SDS and Promega methods were at almost comparable levels whereas the least economical was the Nucleospin 1 and 2 methods. Fig. 1.

**Table 1:** Comparison of concentration, purity and yield of DNA extracted through three commercial kits, SDS and CTAB methods.

Sample weight (mg)	Method	Average DNA concentration ng/µl	Elution volume (µl)	Average Yield (µg)	Yield (ng mg^−^ of wet tissue)	DNA Purity		Duration (h)	Cost/ sample (USD)	Comprehensive cost analysis
						A260/280	A230/260			
200	Nucleospin 1	2.48 ± 0.03^a^	50	0.124 ± 0.15^a^	0.62	1.19 ± 0.00	0.49 ± 0.01	2.5	45.0	0.138
200	Nucleospin 2	3.62 ± 0.31^a^	50	0.181 ± 0.11^a^	0.90	1.45 ± 0.00	1.51 ± 1.12	2.0	45.0	0.110
100	Sigma	1.28 ± 0.02^ab^	50	0.216 ± 0.05^a^	2.16	1.77 ± 0.12	0.06 ± 0.06	1.5	14.25	0.026
500	Promega	2.41 ± 0.21^a^	100	0.241 ± 0.02^a^	0.48	1.38 ± 0.01	0.45 ± 0.05	3.5	13.5	0.058
1500	SDS	7.64 ± 1.50^c*^	2000	15.28 ± 2.99^a^	5.09	1.33 ± 0.05	0.48 ± 0.00	18.0	2.56	0.056
1000	CTAB	6.19 ± 1.65^a^	15,000	92.85 ± 24.8^b**^	92.85	1.85 ± 0.03	1.26 ± 0.10	8.0	2.03	0.019

SDS- Sodium dodecyl sulfate; CTAB- cetyltrimethylammonium bromide. Values superscribed with different letters differ significantly at *p* < 0.01; the Sigma kit cost is 1000 USD for 70 samples. The cost of the Promega kit is 1356.40 USD for 100 samples. The cost of the Nucelospin kit is 2223.10 USD for 50 reactions. DNA concentrations are estimated by Qubit Fluorometer using the dsDNA BR Assay kit. The yield is calculated based on the Qubit reading; Comprehensive-time and cost were calculated as (estimated cost per extraction of any one method/maximum estimated cost among six methods)   x  (estimated time per extraction of any one method/maximum estimated time among six methods) [21].

## PCR amplification with DNA barcoding primers

The universal rbcL  (F- 5′ ATGTCACCACAAACAGAGACTAAAGC 3′; R- 5′ GAAACGGTCTCTCCAACGCAT-3′), trnF (F- 5′CGCGCATGGTGGATTCACAATCC 3′; R- 5′ GTTATGCATGAACGTAATGCTC 3′), and MatK (F- 5′ TAATTTACGATCAATTCATTC’; R- 5′ GTTCTAGCACAAGAAAGTCG 3′) primers were used to check the applicability of the extracted DNA in PCR [22]. All primers were synthesized commercially and purchased from Thermo Fisher Scientific (Waltham, MA, USA), dissolved in 0.1 mM Tris EDTA and adjusted to 1 µM concentration. PCR was performed as per the protocol described elsewhere [5]. In brief, the PCR reaction mixtures (20 µl) contained 1 µl (10 ng) of sample DNA, 4 µl of 5x HOT FIREPol^Ⓡ^ Blend Master mix (Solis BioDyne, Estonia) and 1 µl of each forward and reverse primer (10 µM). The reaction was carried out in Veriti Thermal Cyclers (Applied Biosystems, Grand Island, NY) with initial activation of the DNA polymerase at 95 °C for 12 min, followed by 25 cycles of denaturation at 95 °C for 45 s, annealing at 50–55 °C for 30 s and extension at 70 °C for 30 s. The final extension was carried out at 72 °C for 5 min. The PCR products were visualized on 2.0% agarose gel and ran at 10 V/cm for 1 h. Gel images were captured on a gel documentation system (Chemidoc MP, BioRad, USA).

**Fig. 2:**
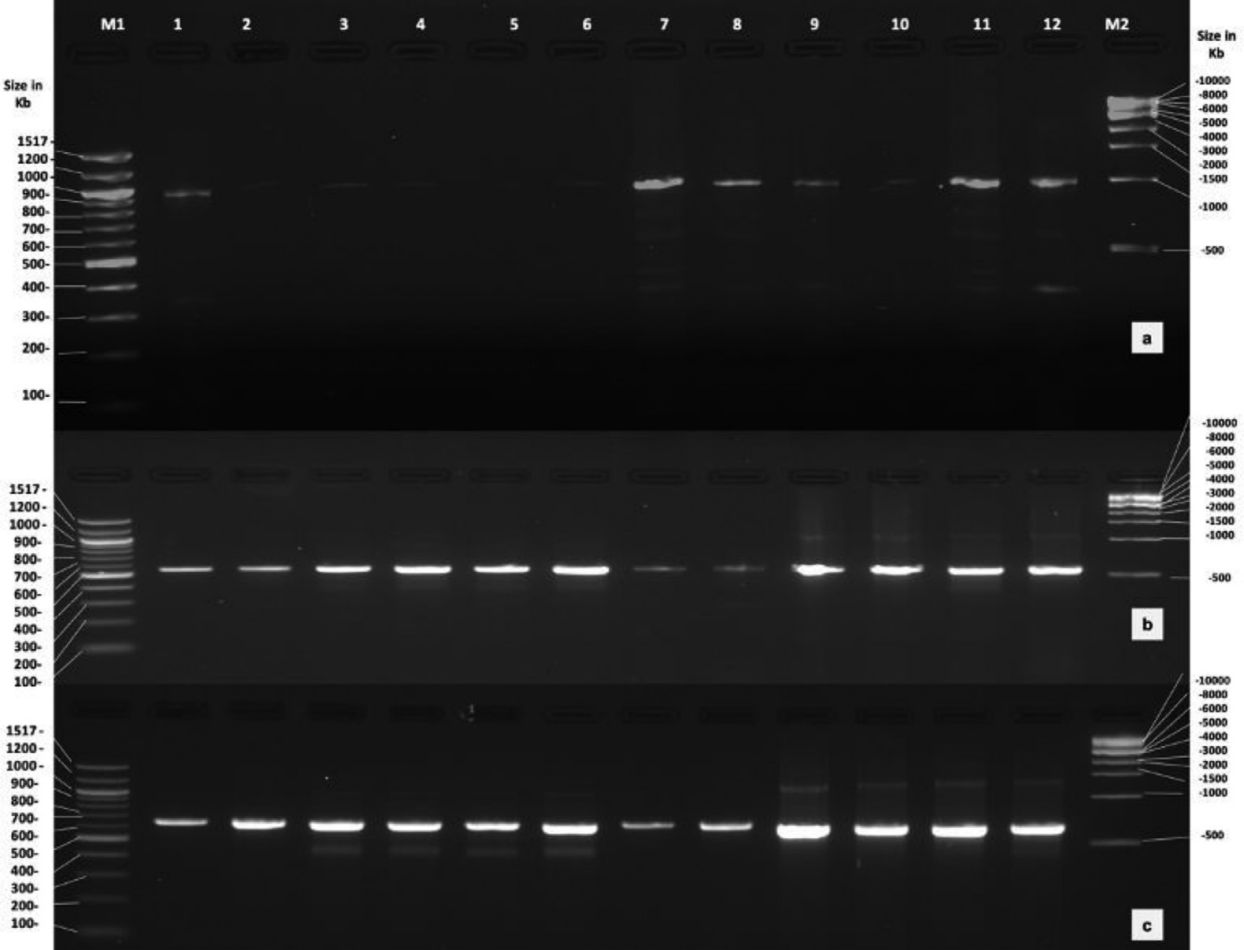
Agarose gel (2.0%) depicting PCR amplification with (a) MatK primers; (b) trnF1 primer; and (c) rbcL 1 primer. M-1 kb marker; Lane 1–2 DNA extracted by Sigma kit; Lane 3–4 DNA extracted by Nucleospin I; Lane 5–6 DNA extracted by Nucleospin 2; Lane 7–8 DNA extracted by Promega kit; Lane 9–10 DNA extracted by SDS method; Lane 11–12 DNA extracted by CTAB method.

PCR with trnF1, rbcL1 and matK primers amplified a fragment of 510, 765 and 983 bp respectively, (Fig 2a–c) in DNA isolated by all the methods, however clear intact bands were visualized only in DNA extracted by CTAB.

## Next-generation sequencing (NGS)

**Fig. 3:**
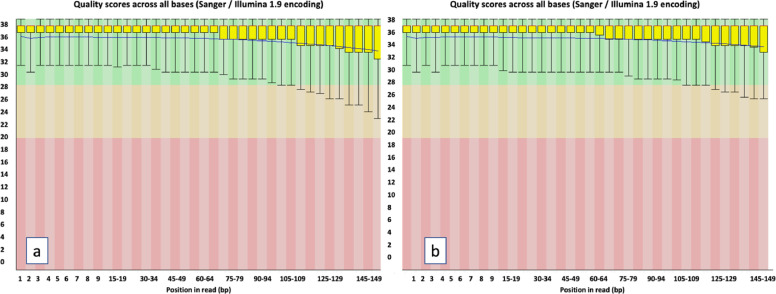
Paired-end read quality (PHRED score) distribution of *Acacia pachyceras* samples sequenced on an Illumina HiSeq 2500 platform (a) Read 1 (b) Read 2. For each position, a box-whisker plot is drawn. Redline—median value, yellow box—inter-quartile range (25–75%), upper and lower whiskers—10 and 90% points, blue line—mean quality.

Approximately, 1 µg of DNA isolated by the CTAB method was lyophilized and shipped to the Beijing Genomics Institute (BGI), Hongkong for high throughput sequencing [10]. After initial quality checks, the DNA was processed for library preparation (2 X 150 paired-end) and sequenced on an Illumina HiSeq 2500 platform. Approximately, 109 GB of raw data were generated. Quality parameters for the raw data were accessed through FASTQC [23]. The raw reads were trimmed using the Trimmomatic v. 0.17 [24], [25]. Read quality distributions based upon PHRED quality scores, ranged between 34.0–37.0 (mean-36.0). A base call accuracy of approximately 99.9%, favorably desirable for big data sequencing applications was obtained (Fig. 3a,b).

## Declaration of Competing Interest

The authors declare that they have no known competing financial interests or personal relationships that could have appeared to influence the work reported in this paper.

## Appendix Supplementary materials

Image, application 1
