# Supplementary material for: Comparison and optimization of DNA Isolation protocols for high throughput genomic studies of Acacia pachyceras Schwartz
Source: MethodsX. 2022 Jul 28;9:101799. doi: 10.1016/j.mex.2022.101799 (PMC9386096; doi:10.1016/j.mex.2022.101799)
Supplement: Supplementary file 1 [file mmc1.docx]

**Supplementary File: Reagents and Cost Analysis**

**Table S1 Reagents required for the SDS protocol and their cost breakdown**

| **Reagent** | **Make** | **Stock** | **Amount taken to prepare the stock** | **Price in USD** | **Cost per ml or per g** | **Cost per sample** |
| --- | --- | --- | --- | --- | --- | --- |
| Tris | Gibco BRL, ultra pure, Life Technologies, CA, US | 1M | 121.4/1000ml | 60 (1kg) | 7.284 | 0.021 |
| EDTA | Sigma Aldrich, NY, USA | 0.5M | 186.1g/1000ml | 18 (500 g) | 6.6996 | 0.020 |
| Proteinase K (10mg/ml) | Qiagen, MD, USA | 10mg/ml | 10ul | 90 (1.25 ml) | 0.72 | 0.720 |
| SDS | Gibco BRL, ultra pure, Life Technologies, CA, US | 20% | 20g/100ml | 75 (1 kg) | 1.5 | 0.045 |
| Potassium acetate | Sure Chem products, England | 5M | 294.42/1000ml | 9 (500 g) | 5.29956 | 0.063 |
| Isopropanol | Carlo Erba reagents, France | 10 ml | 10ml | 15 (2.5L) | 0.006 | 0.006 |
| Ethanol | Merck, Honeywell Raedel-de-Haen france | 20 ml | 20 ml | 18 (2.5L) | 0.0144 | 0.011 |
| PVP | Sigma Aldrich, Germany | 2g | 2g | 25 (100 g) | 0.5 | 0.500 |
| Hydrochloric Acid | Merck, Darmstadt, Germany | 20mM | 222.3g/1000ml | 16.5 (2.5L) | 3.7791 | 0.022 |
| Potassium metabisulfite | BDH Laboratories, England | 50mM | 15.45/1000ml | 7.95 (113g) | 618 | 0.927 |
| DTT | Sigma Aldrich, Germany | 500mM | 292g/1000ml | 80 (2 g) | 3.504 | 0.052 |
| NaCl | BDH Laboratories, England | 500mM | 292g/1000ml | 6 (500 g) | 3.504 | 0.052 |
| TE |  | 1ml Tris + 0.2 ml 0.5M EDTA | 2 ml | 9 (500 g) | 8.62392 | 0.172 |
|  |  | **Total Cost per sample in USD** | | | | **2.56** |

**Table S2 Reagents required for the CTAB protocol and their cost breakdown**

| **Reagent** | **Make** | **Stock** | **Amount taken to prepare the stock** | **Price in USD** | **Cost per ml or per g** | **Cost per sample** |
| --- | --- | --- | --- | --- | --- | --- |
| CTAB | Bioworld Fine Research Chemicals, OH, USA | 10% | 10g/100ml | 270 (100g) | 27.0 | 0.500 |
| Tris-Cl | Gibco BRL, ultra pure, Life Technologies, CA, US | 1M | 121.4/1000ml | 60 (1kg) | 7.284 | 0.021 |
| NaCl | BDH Laboratories, England | 5M | 292g/1000ml | 6 (500 g) | 3.504 | 0.010 |
| EDTA | Sigma Aldrich, NY, USA | 0.5M | 186.1g/1000ml | 18 (500 g) | 6.6996 | 0.003 |
| 2-mercaptoethanol | Merck, Darmstadt, Germany | 20 ul | 0.00009/ul | 45 (500 ml) | 0.0018 | 0.002 |
| Chloroform | Fisher Scientific, UK | 12 ml | 12ml | 21 (2.5L) | 0.01008 | 0.010 |
| Isoamyl alcohol | Merck, Darmstadt, Germany | 0.5 ml | 0.5ml | 75 (2.5L) | 0.0015 | 0.002 |
| Isopropanol | Carlo Erba Reagents, Val-de-Reuil, France | 10 ml | 10ml | 15 (2.5L) | 0.006 | 0.006 |
| Ethanol | Merck, Honeywell Raedel-de-Haen France | 20 ml | 20 ml | 18 (2.5L) | 0.0144 | 0.011 |
| RNase A | Qiagen, USA | 10ul | 10 ul | 113 (2 ml) | 0.565 | 0.565 |
| TE |  | 1ml Tris + 0.2 ml 0.5M EDTA | 10 ml | 9 (500 g) | 8.62392 | 0.862 |
|  |  | T**otal cost per sample** | | | | **2.03** |
